# Discontinuous Shear Thickening in Biological Tissue Rheology

**DOI:** 10.1103/physrevx.14.011027

**Published:** 2024-02-22

**Authors:** Michael J. Hertaeg, Suzanne M. Fielding, Dapeng Bi

**Affiliations:** 1 Department of Physics, Durham University, Science Laboratories, South Road, Durham DH1 3LE, United Kingdom; 2 Department of Physics, Northeastern University, Massachusetts 02115, USA

**Keywords:** Biological Physics, Soft Matter, Statistical Physics

## Abstract

During embryonic morphogenesis, tissues undergo dramatic deformations in order to form functional organs. Similarly, in adult animals, living cells and tissues are continually subjected to forces and deformations. Therefore, the success of embryonic development and the proper maintenance of physiological functions rely on the ability of cells to withstand mechanical stresses as well as their ability to flow in a collective manner. During these events, mechanical perturbations can originate from active processes at the single-cell level, competing with external stresses exerted by surrounding tissues and organs. However, the study of tissue mechanics has been somewhat limited to either the response to external forces or to intrinsic ones. In this work, we use an active vertex model of a 2D confluent tissue to study the interplay of external deformations that are applied globally to a tissue with internal active stresses that arise locally at the cellular level due to cell motility. We elucidate, in particular, the way in which this interplay between globally external and locally internal active driving determines the emergent mechanical properties of the tissue as a whole. For a tissue in the vicinity of a solid-fluid jamming or unjamming transition, we uncover a host of fascinating rheological phenomena, including yielding, shear thinning, continuous shear thickening, and discontinuous shear thickening. These model predictions provide a framework for understanding the recently observed nonlinear rheological behaviors *in vivo*.

## I. INTRODUCTION

During embryonic morphogenesis, biological tissues undergo dramatic deformations in order to form functional organs. The tissues of mature organisms likewise continually suffer stresses and deformations. The success of embryonic development and the maintenance of proper physiological functioning accordingly both depend intimately on a tissue’s rheological (deformation and flow) properties [1]. On short timescales, tissues can withstand mechanical stresses. Over longer timescales, they remodel via cell neighbor exchanges (topological T1 transitions) [2–5], which thus constitute a key rate-limiting step in important processes such as embryo development, wound healing, and cancer metastasis. Recent evidence further suggests that dense confluent tissues, which have no gaps between cells, are poised in the vicinity of a transition between a jammed, solidlike state and an unjammed, fluidlike state [6–14].

From a fundamental viewpoint, mechanical stresses can either originate *internally* within a biological tissue, via spontaneously active processes intrinsic to the cellular level, such as cell contractility [15,16], polarized motility [17], or mitosis [18,19]; or they can be exerted *externally*, by surrounding tissues and organs [20,21]. Recent experiments have shown that cell collectives subjected to externally applied stretching [22–25] or shear [26] deformations show a strongly nonlinear rheological response. Tissues deformed by internally active stresses at the cellular level have likewise been seen to exhibit extreme mechanical phenomena such as fracturing [27].

Perhaps surprisingly, studies of tissue mechanics to date have largely been confined *either* to the response of tissues to externally imposed stresses *or*, separately, to phenomena arising from internally active processes. Crucially, however, most living tissues exist in a state where *both* forms of driving work together in concert. For example, during Drosophila embryogenesis, polarized actomyosin contractility at the single-cell level interacts with external stresses exerted by neighboring tissues to cause the tissue to flow plastically in convergent extension [21,28]. During cancer progression, tumor-cell collectives constantly experience mechanical stimuli such as compression and shear stresses from the surrounding extracellular matrix (ECM) [29]. At the same time, tumor cells generate actomyosin contractility at the single-cell level. This interplay between external microenvironmental stresses and internal motility has been shown to be central to determining whether a cell cluster is jammed or unjammed [12]. Recent work on cancer migration also suggests that tumor fluidity depends not only on the single-cell invasive potential (akin to our activity) but also on the compressive and shear stresses they experience due to the ECM [30].

With these motivations, in this work, we elucidate, in particular, the way in which the interplay between globally *external* and locally *internal* active driving determines the emergent mechanical properties of the tissue as a whole. Model predictions point towards a framework for understanding the recently observed range of nonlinear rheological behaviors *in vivo* [27,28,31] and *in vitro* [23,26]. For a tissue in the vicinity of a solid-fluid jamming or unjamming transition, we uncover a host of fascinating rheological phenomena, including yielding, shear thinning, continuous shear thickening (CST), and discontinuous shear thickening (DST).

Beyond this context of biological tissues, shear thickening has been the focus of intense recent research in the rheology literature more broadly because of its widespread occurrence in dense granular materials and suspensions [32–35]. Indeed, simulations [36,37] and experiments [38] on dense suspensions show a large discontinuous increase in viscosity with increasing shear rate, attributed to a crossover between hydrodynamic and frictional interparticle interactions. For shear rates in this transition region, large stress fluctuations are seen, with an intermittent bimodal switching between low and high viscosity branches of the flow curve [32,37]. Associated with this shear thickening transition is the formation of bands of different shear stress stacked with layer normals in the vorticity direction [39]. Our prediction of DST in biological tissues suggests that this phenomenon may be present in a broader class of materials than is evident from this existing rheology literature.

## II. MODEL

The vertex model that we simulate represents the tightly packed cells of a 2D tissue monolayer as a tiling of n=1⋯N polygons, defined by the positions of the polygon vertices [13,40–44]. The vertices of any polygon are joined by edges that form the boundaries with the adjoining cells. Each vertex is shared by three cells and each edge by two cells.

The elastic energy of the vertex model comprises two contributions. The first is set by the deviation of the area A of a cell from a target value $A_{0}$, providing a 2D toy model of 3D cell volume incompressibility. The second contribution is set by the deviation of the cell perimeter P from a target value $P_{0}$. Summing over all cells in the packing, the energy is then

(1) $$E=\frac{1}{2}\sum_{n=1}^{N}\;\left[\kappa_{A}{\left(A_{n}-A_{n0}\right)}^{2}+\kappa_{P}{\left(P_{n}-P_{n0}\right)}^{2}\right].$$

The quantity $p_{0}=P_{n0}/\sqrt{A_{n0}}$ defines a cell shape factor and is an important control parameter in our study. We set it the same value for all cells in any simulation, independent of n. In physical terms, $p_{0}$ is commonly attributed to a competition between cell cortical contractility and cell-cell adhesion [42,45–48], although recent experiments also imply a relationship with cell-substrate traction [49]. Cell shape has been shown experimentally to predict jamming behavior in epithelial tissues [6,50]. The elastic constants $\kappa_{P}$ and $\kappa_{A}$ set the strength of the perimeter and area interactions, and we choose $\kappa_{P}=1$ as our basic unit of stress.

**FIG. 1.**
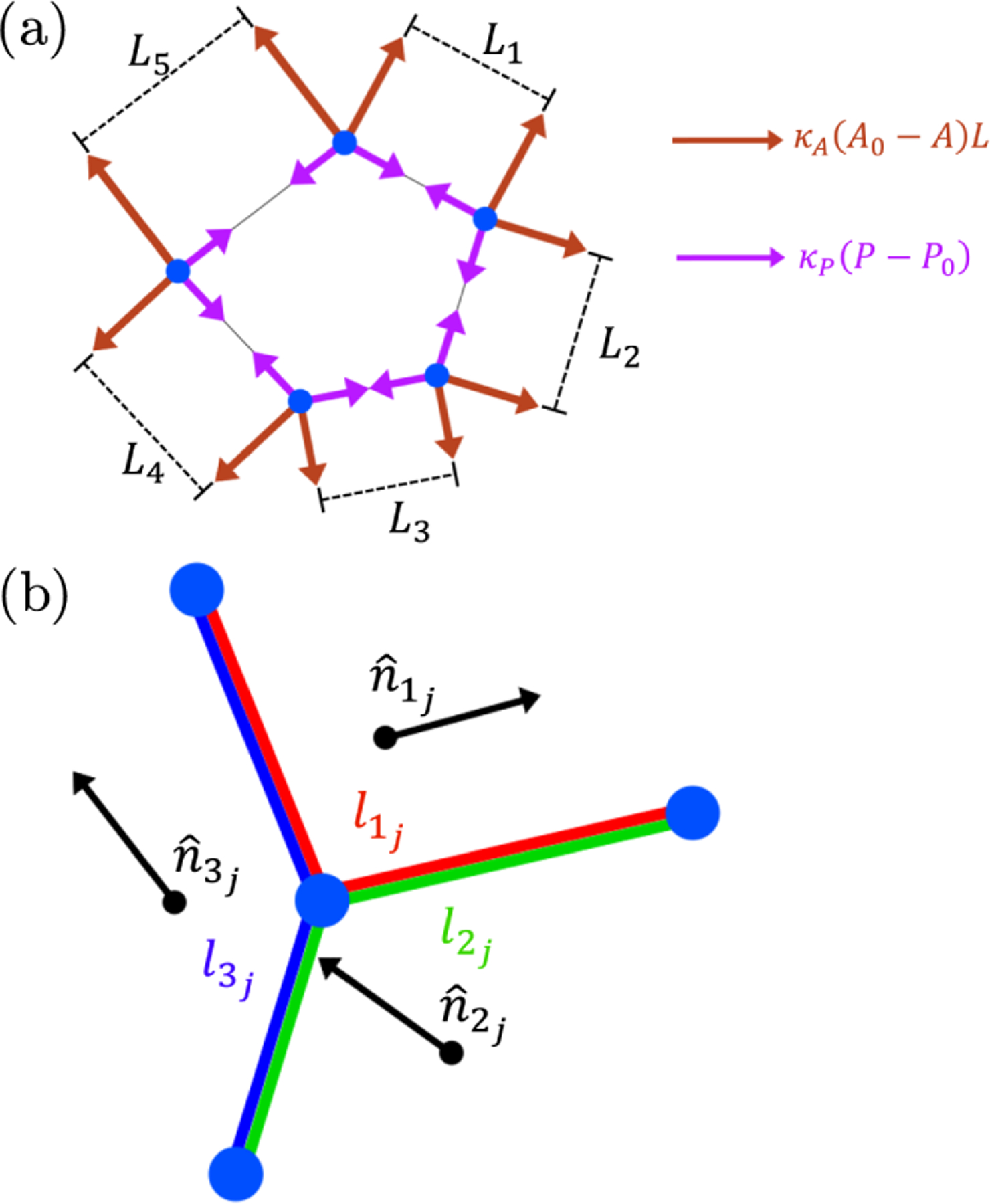
(a) Diagram of elastic forces in the vertex model. Forces tangential to cell edges are proportional to the deviation in cell perimeter and are all of the same magnitude for a single cell. Forces perpendicular to edges are proportional to both the deviation in cell area and the associated edge length. (b) Diagram of the jth vertex in the packing (central blue circle), showing the three edges connecting this vertex to its three neighboring vertices (other blue circles). The cell polarization vectors ${\overset{ˆ}{n}}_{i_{j}}$ of the three adjoining cells $i_{j}=1,2,3$ are shown as vectors. The associated lengths $l_{i_{j}}$ used in the weighted sum to calculate the polarization vector of the vertex are shown by colored lines.

The elastic forces exerted on the vertices of any cell due to the elastic contributions of that cell are sketched in Fig. 1(a). In this sketch, consider a representative edge of length L, connecting two representative adjacent vertices. The cell that is sketched then contributes to each of these two vertices an equal and opposite tensionlike force of magnitude $\kappa_{P}\left(P-P_{0}\right)$, acting tangentially along the edge, inwards along the edge when $P>P_{0}$, and outwards when $P<P_{0}$. The cell shown also contributes to the same two vertices a pressurelike force of magnitude $\kappa_{A}\left(A-A_{0}\right)L$, acting perpendicularly to the edge, in towards the cell when $A>A_{0}$, and outwards when $A<A_{0}$. These expressions are derived in the Appendix. Each vertex in Fig. 1(a) additionally belongs to two further cells (not shown) that contribute corresponding elastic forces. The total elastic force ${\vec{F}}_{j}$ on the jth vertex in the tiling is calculated by summing these contributions from its three shared cells.

In an externally applied simple shear flow of rate $\dot{\gamma }$, with flow direction x and flow-gradient direction y, the position ${\vec{r}}_{j}$ of the jth vertex in the tiling obeys overdamped dynamics with drag coefficient ζ:

(2) $$\frac{d{\vec{r}}_{j}}{dt}=\frac{1}{\zeta }\left({\vec{F}}_{j}+v\sum_{i_{j}=1}^{3}\;\;w_{i_{j}}{\overset{ˆ}{n}}_{i_{j}}\right)+\dot{\gamma }y_{j}\overset{ˆ}{x},$$

with Lees-Edwards periodic boundary conditions [51].

The second term on the right-hand side of this equation describes a random motile activity [5,52,53]. The magnitude v of this activity is an important control parameter in our study. The direction of the motility of the jth vertex in the tiling is prescribed by the weighted sum of the polarization vectors ${\overset{ˆ}{n}}_{i_{j}}=\left(\text{cos}\theta_{i_{j}},\text{sin}\theta_{i_{j}}\right)$ of the three cells $i_{j}=1,2,3$ in contact with that jth vertex. The polarization angle of each cell in the tiling is initialized randomly at the start of any simulation from a uniform distribution in the range 0 to 2π. It thereafter experiences angular diffusion with a diffusion coefficient $D_{r}$, modeled via Gaussian random noise. Accordingly, the polarization angle of the nth cell in the tiling obeys

(3) $$\frac{d\theta_{n}}{dt}=\eta_{n},$$

in which $\eta_{n}$ is a random variable with statistics [42]

(4) $$\left\langle \eta_{n}(t)\right\rangle =0,\;\left\langle \eta_{n}(t)\eta_{m}\left(t^{\prime }\right)\right\rangle =2D_{r}\delta_{nm}\delta \left(t-t^{\prime }\right).$$

The weighting factors $w_{i_{j}}$ in Eq. (2) ensure that the largest contribution to the polarization vector of our representative vertex (the jth in the tiling) arises from whichever of its three associated cells $i_{j}=1,2,3$ has the largest value of the summed lengths of cell edges that contact that vertex. Specifically, we define $l_{i_{j}}$ to be the summed length of the two edges of the $i_{j}$th cell in contact with vertex j, as shown by the color-coded lines in Fig. 1(b), and set

(5) $$w_{i_{j}}=\frac{l_{i_{j}}}{12L_{Tj}},$$

consistent with the weighting function used in previous work [5,13]. Here, $L_{Tj}$ is the total length of the three edges in contact with vertex j. Topological T1 cell-cell rearrangement events also intermittently arise, leading to plastic stress relaxation. Specifically, when any cell edge length becomes smaller than a threshold value $l_{T1}$, a T1 event occurs. Prior to a T1, the selected edge is defined by two vertices, one shared between cells αβγ and the other αβδ. The T1 event then replaces these two old vertices with two new ones, shared by cells αγδ and βγδ [13,54].

To initialize an amorphous cellular tiling, we start from a uniform lattice of monodisperse hexagonal cells of cell edge length 1 stacked in $\sqrt{N}$ rows, each of $\sqrt{N}$ cells. Target perimeter and area values are then assigned to each cell. To avoid the effects of crystallization associated with monodisperse packings [55], we use a bidisperse packing in which half the cells have a smaller size and half a larger size. Specifically, we assign these two populations target perimeters in the ratio 1:1.4, respectively. To maintain a consistent target shape factor $\left(p_{0}=\left(P_{0}/\sqrt{A_{0}}\right)\right)$ between these two populations, their target areas are set in the ratio $1:1.4^{2}$, respectively. The overall scale of the target area is set such that the target area summed over all cells equals that of the domain size created in the initial uniform hexagonal tiling. The packing is then randomized by implementing cell motility with nonzero $v=v_{\text{prep}}$ and $D_{r}=D_{r,\text{prep}}$ in the absence of shear for $t_{\text{prep}}$ time units, then subsequently relaxing the system with zero activity (and zero shear) for $t_{\text{relax}}$ time units. Choosing $v_{\text{prep}}=4.0,\;D_{r,\text{prep}}=0.25,t_{\text{prep}}=14.5,t_{\text{relax}}=5.5$ produces a random bidisperse initial cellular tiling with fully relaxed cell areas and perimeters.

The equations of motion described above are integrated using the explicit Euler method with time step dt, both during the preparation stage just discussed and the subsequent shearing stage. The time step dt is converged to the limit dt→0, and the system size is converged to the limit N→∞.

The values, symbols, and dimensions for the parameters of the model and shear protocol are listed in Table I.

In what follows, we report the steady-state shear stress σ in the tissue. For any individual cell with vertices numbered c=1⋯C, a cell level stress is calculated at any time as [56]

(6) $$S_{\alpha \beta }=\frac{1}{A}\sum_{c=1}^{C}\;F_{c\alpha }r_{c\beta }.$$

Here, $F_{c\alpha }$ is the α component of the elastic force on vertex c from the elastic contributions of that single cell, $r_{c\beta }$ is the β component of the position of vertex c, and A is the cell’s area. These individual cell stresses, thus defined, are then averaged over all cells in the packing. The packingaveraged shear stress is then averaged over many strain units once a state of statistically steady shear is attained and, furthermore (for each set of model parameters), over three runs with different random number of seeds. It is this averaged shear stress that is reported in the results that follow. We have checked it to be robust to changes in system size for N>100. Fluctuations about the average decrease with increasing N.

In the absence of internal activity (v=0) and external applied shear $\left(\dot{\gamma }=0\right)$, the vertex model captures a fluid-solid transition at a critical target cell shape $p_{0}=p_{0}^{*}$ [41,42,48,57], with $p_{0}^{*}\approx 3.81$ for the bidisperse tiling studied here. For $p_{0}<p_{0}^{*}$, cells cannot attain their target shape, and the energy barriers to local T1 cell rearrangements are significant: The tissue resists shear, giving a solid phase. For $p_{0}>p_{0}^{*}$, cells achieve their target shape, and the energy barriers to rearrangements are small, resulting in a liquid phase that cannot resist shear [48]. A nonlinear shear applied quasistatically $\left(\dot{\gamma }\to 0\right)$, however, induces a solidification transition at a critical strain $\gamma_{c}\left(p_{0}\right)$ for $p_{0}^{*}<p_{0}<p_{0}^{**}$, with $p_{0}^{**}\approx 4.03$ [58]. It does so by deforming cells such that they can no longer attain their target shape, eliminating the zero-shear liquid and inducing a solidlike response. The steady-state flow curve of shear stress vs shear rate, $\sigma (\dot{\gamma })$, then displays a yield stress $\sigma_{Y}={lim}_{\dot{\gamma }\to 0}\;\sigma (\dot{\gamma })\neq 0$ for all $p_{0}<p_{0}^{**}$ [58].

## III. RESULTS

**FIG. 2.**
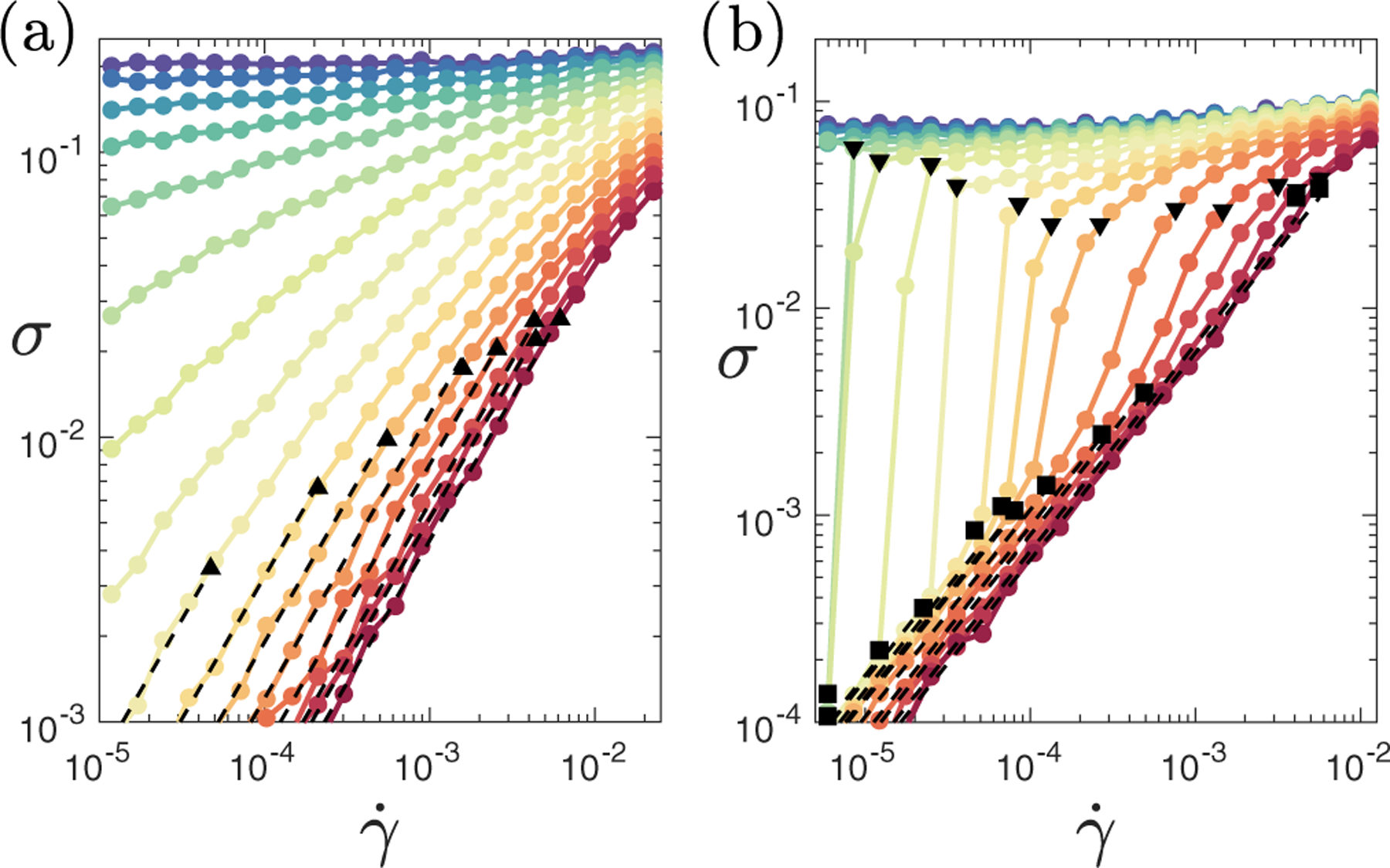
Steady-state flow curves of shear stress as a function of shear rate. (a) For fixed $p_{0}=3.65$ in the (zero-activity, zero-shear) solid phase with activity v=0.0,0.1,0.2,0.3,0.4,0.5,0.6,0.7,0.8,0.9,1.0,1.1,1.2,1.3,1.4,1.5 (from top to bottom). For low activity, we find a yield stress in the limit of low strain rate. For high activity, we see Newtonian flow response at low strain rates with shear thinning for higher strain rates. (b) For a fixed $p_{0}=3.90$ in the (zero-activity, zero-shear) liquid phase with activity v=0.00,0.01,0.02,0.03,0.04,0.05,0.06,0.08,0.10,0.12,0.14,0.16,0.20,0.25,0.30,0.35,0.40 (from top to bottom). With no activity, we find a yield stress in the limit of low strain rate. With modest levels of activity, Newtonian flow response at low strain rate gives way to a discontinuous shear thickening transition with increasing shear rate. Dashed lines fit regimes of constant viscosity, $\eta \left(p_{0},v\right)=\sigma /\dot{\gamma }$. Black squares show ${\dot{\gamma }}_{\text{thick}}$ and triangles ${\dot{\gamma }}_{\text{thin}}$, defined in the main text.

We start by exploring the effects of activity on a sheared tissue, reporting in Fig. 2(a) steady-state flow curves $\sigma (\dot{\gamma })$ in the (zero-activity, zero-shear) solid phase, $p_{0}<p_{0}^{*}$. At zero activity, we see a yield stress $\sigma_{Y}={lim}_{\dot{\gamma }\to 0}\;\sigma (\dot{\gamma })\neq 0$, indicating a solidlike response with infinite viscosity $\eta =\sigma /\dot{\gamma }$ in quasistatic shear $\dot{\gamma }\to 0$, consistent with Ref. [58]. In contrast, at high activity, we find liquidlike flow with $\sigma =\eta \dot{\gamma }$, in the limit of small shear rate $\dot{\gamma }\to 0$, which is termed Newtonian flow behavior. The viscosity $\eta =\eta \left(p_{0},v\right)$ is fit by the black dashed lines.

**FIG. 3.**
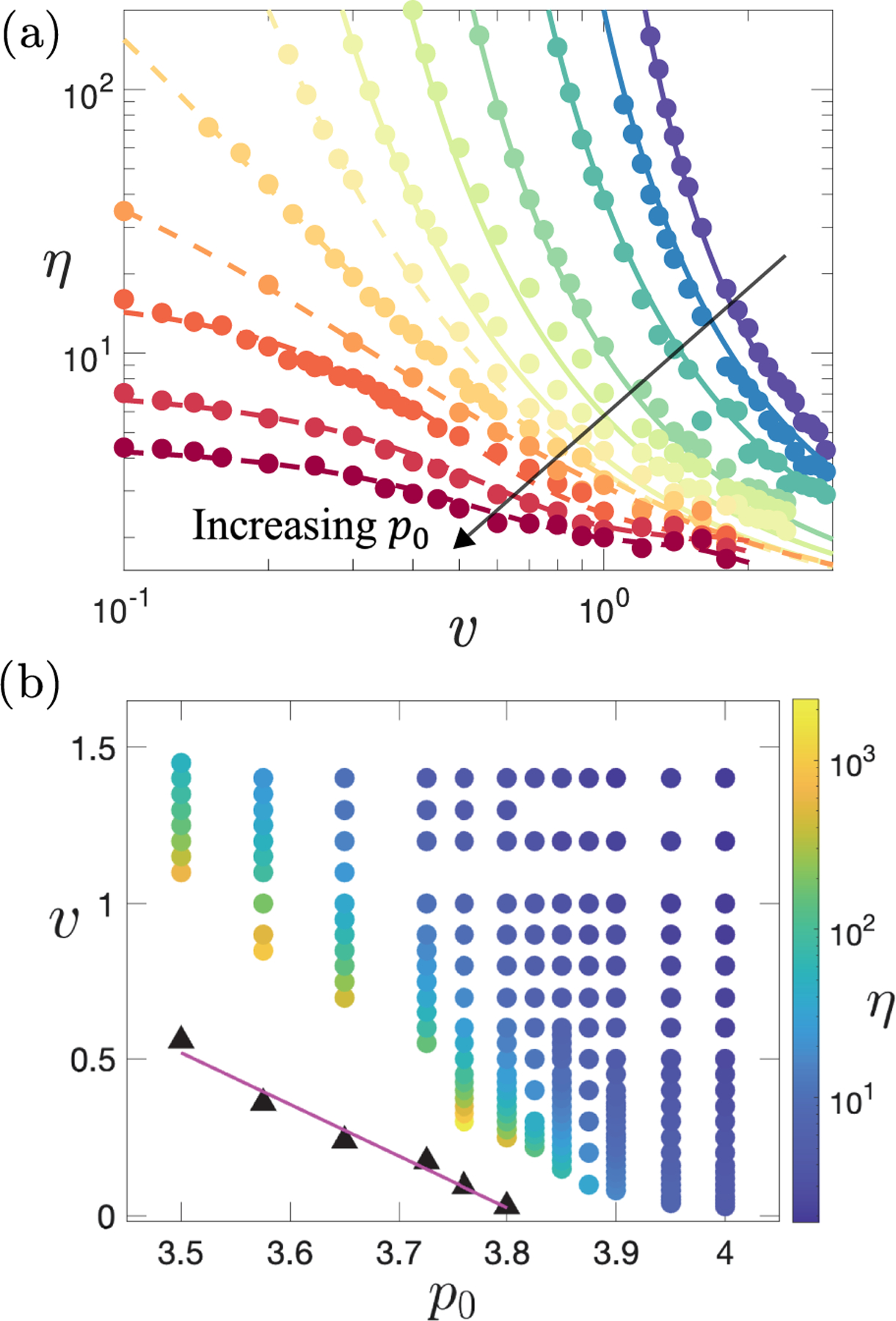
Newtonian viscosity $\eta \left(p_{0},v\right)=\sigma /\dot{\gamma }$ from black-dashed fits in Fig. 2. (a) Plotted vs activity v for target shape $p_{0}=3.500,\;3.575,3.650,3.725,3.760,3.800,3.825,3.850,3.875,3.900,\;3.950,4.000$ (from top to bottom). Solid lines are fits to the VFT form $\eta \sim exp\left[1/\left(v-v_{c}\right)\right]$ for $p_{0}<p_{0}^{*}\approx 3.81$, suggesting a viscosity divergence as $v\to v_{c}\left(p_{0}\right)$. Dashed lines are spline fits for $p_{0}>p_{0}^{*}$. (b) Color map in the plane of v and $p_{0}$. Black triangles show the value of $v=v_{c}\left(p_{0}\right)$ at which the VFT fit predicts the viscosity to diverge. The magenta line is the linear fit to the black triangles.

The zero-shear $\left(\dot{\gamma }\to 0\right)$ viscosity $\eta \left(p_{0},v\right)$ in the (zero-activity, zero-shear) solid phase, $p_{0}<p_{0}^{*}$, thus increases dramatically with decreasing activity v at fixed target cell shape. In Fig. 3(a), we fit this increase to the Vogel-FulcherTamman (VFT) form $\eta \sim exp\left[1/\left(v-v_{c}\right)\right]$ to find the critical activity $v=v_{c}\left(p_{0}\right)>0$ below which η diverges at any $p_{0}<p_{0}^{*}$. This divergence of the zero-shear viscosity at a critical $v_{c}\left(p_{0}\right)$ for $p_{0}<p_{0}^{*}$ may indicate a true yield stress $\sigma_{Y}$ for all $0\leq v<v_{c}$, consistent with that at v=0 [58], although a power law $\sigma \propto {\dot{\gamma }}^{n}$ with n<1 is not ruled out for $0<v<v_{c}$. Either way, in the solid phase, $p_{0}<p_{0}^{*}$, a critical activity $v_{c}\left(p_{0}\right)$ is needed to eliminate solidlike behavior in favor of Newtonian flow with finite η. This critical $v_{c}$ is plotted vs $p_{0}$ in Fig. 3(b), which also shows a color map of η in the plane of $v,p_{0}$. The zero-shear viscosity is also consistent with the viscosity calculated based on the Green-Kubo relation [59]. A linear fit suggests that $v_{c}$ falls to zero at the (zero-activity, zero-shear) solid-liquid transition $p_{0}=p_{0}^{*}$. This intercept is consistent with similar data in linear studies [59]. However, the curve shape differs, implying a different mechanism at nonzero activity.

The flow curves just discussed, for a tissue in its (zero-activity, zero-shear) solid phase, closely resemble those of complex fluids such as glassy colloidal and jammed athermal soft particle suspensions [60–62]. These curves show a yield stress at high packing fraction ϕ and low temperature, analogous to our curves for low activity. Particle suspensions also show low-shear Newtonian behavior at low ϕ and high temperature, analogous to ours at high activity.

Next, we consider the effect of activity on a sheared tissue in its liquid phase, $p_{0}>p_{0}^{*}$. See the steady-state flow curves in Fig. 2(b). In notable contrast to the solid phase, these curves closely resemble the flow curves of dense frictional suspensions and granular matter [63–65]. In particular, they show DST, in which the shear stress jumps discontinuously with increasing strain rate at high ϕ (in suspensions) or low activity (here). DST then gives way, at lower ϕ (in suspensions) or higher activity (here), to CST, in which the stress still steepens with shear rate, but without jumping.

**FIG. 4.**
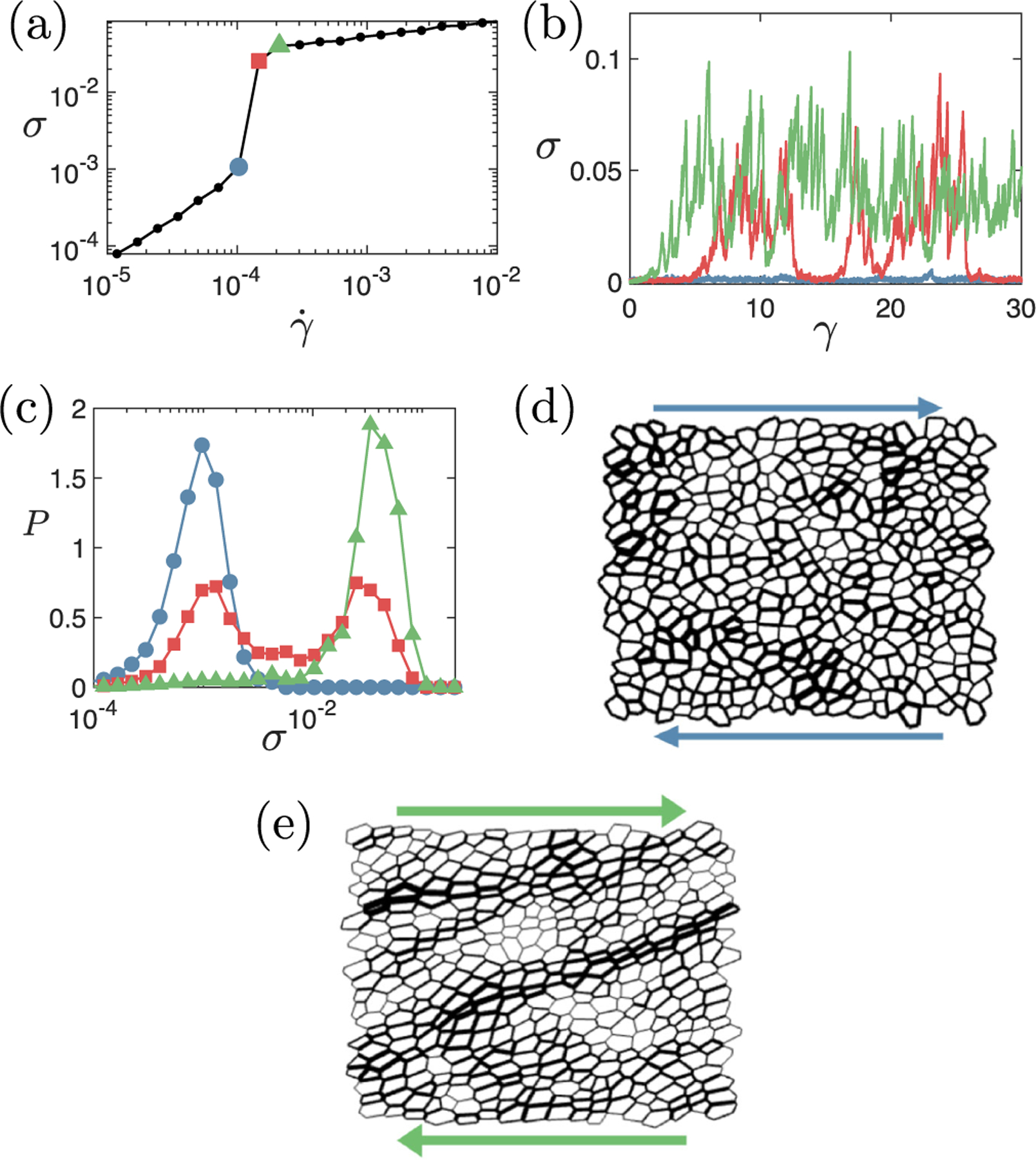
Exploring the DST transition. (a) Representative flow curve showing the DST transition. (b) Stress as a function of strain $\gamma =\dot{\gamma }t$ for the three imposed strain rates denoted by shapes of corresponding color in panel (a): blue circles, $\dot{\gamma }=5.14\times 10^{-5}$; red squares, $\dot{\gamma }=7.36\times 10^{-5}$; green triangles, $\dot{\gamma }=1.1\times 10^{-4}$. (c) Corresponding probability distributions of the logarithm of the stress. Representative state snapshots at (d) $\dot{\gamma }=5.14\times 10^{-5}$ (blue circles) and (e) $\dot{\gamma }=1.1\times 10^{-4}$ (green triangles), with the line thickness of any cell edge proportional to the tensile stress it carries. Regions of high stress are distributed through the system in panel (d) but formed into system-spanning force chains in panel (e). Target cell shape $p_{0}=3.9$, and activity v=0.12.

We next explore the origins of DST by examining the spatial-temporal evolution of the stress for different shear rates at fixed activity and $p_{0}$. An example flow curve for $v=0.12,p_{0}=3.90$ is shown in Fig. 4(a). In Fig. 4(b), the stress as a function of strain $\gamma =\dot{\gamma }t$ (which is proportional to time t, given constant $\dot{\gamma }$) is plotted for three different shear rates, with line colors corresponding to marker colors in Fig. 4(a). The corresponding stress distributions over each of these strain series are shown in Fig. 4(c). For a low-shear rate (blue), the stress fluctuates modestly (in proportional terms) around a low value. Similarly, for a high-shear rate (green), the stress fluctuates modestly around a high value. In contrast, at an intermediate-shear rate (red), the stress intermittently switches between these low and high stress states to give a bimodal distribution.

This intermittent, bimodal stress evolution at the DST transition is also seen in frictional suspensions, where it is caused by percolating compressive force chains [32–35,63,66,67]. Our simulations likewise evidence percolating force chains in the vertex model of biological tissue: Figures 4(d) and 4(e) show representative state snapshots corresponding to the lowest and highest strain rates in Figs. 4(a) and 4(b), with the thickness of each cell edge proportional to the tensile stress it carries. In the low stress (unthickened) state, the tension is distributed fairly evenly across the system. In contrast, the high stress (thickened) state displays system-spanning force chains. In important contrast to the *compressive* force chains that form in frictional suspensions, however, we find these stresses to be tensile in nature in tissues. This finding is consistent with recent computational [10,15,68] and experimental [6,69] studies, which indeed found that tensile stresses overwhelmingly dominate in biological tissues.

To further characterize the regime of shear thickening, we define two characteristic shear rates, each via the logarithmic slope of the flow curve, $G=dlog\sigma /dlog\dot{\gamma }.\;(G=1$ indicates Newtonian behavior.) First, we define the onset of shear thickening in Fig. 2(b) via the shear rate ${\dot{\gamma }}_{\text{thick}}$ (shown by black squares) at which G first increases above 1+ϵ, with ϵ=0.2. Second, we define the reversion to shear thinning at higher strain rates via the shear rate ${\dot{\gamma }}_{\text{thin}}$ (black triangles) at which G first falls below 1-δ, with δ=0.1. At low activity, where DST arises, ${\dot{\gamma }}_{\text{thin}}={\dot{\gamma }}_{\text{thick}}$, to within the resolution of $\dot{\gamma }$ values simulated.

**FIG. 5.**
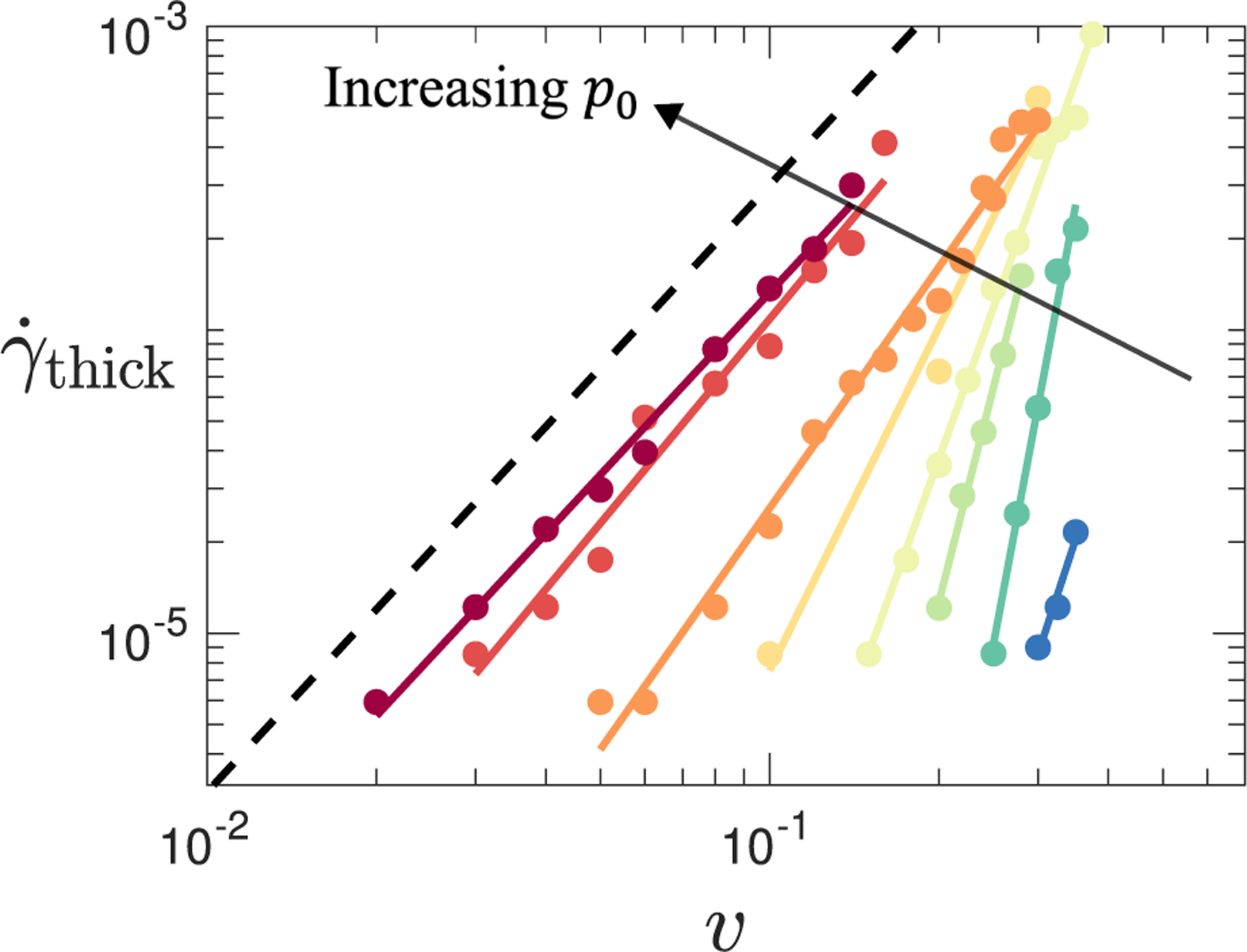
Shear rate ${\dot{\gamma }}_{\text{thick}}$ at the onset of shear thickening as a function of activity for target cell shape $p_{0}=3.760,3.800,\;3.825,3.850,3.875,3.900,3.950,4.00$ (from left to right). Colored straight lines show power-law fits to data, ${\dot{\gamma }}_{\text{thick}}\propto v^{\alpha }$, implying that shear thickening will be present even at very low levels of activity. The dotted black line shows the power α=2 as a guide to the eye.

Figure 5 shows ${\dot{\gamma }}_{\text{thick}}$ as a function of activity v for several values of the target shape $p_{0}$. For $p_{0}$ values comfortably inside the (zero-activity, zero-shear) fluid phase above $p_{0}^{*}$, we find ${\dot{\gamma }}_{\text{thin}}\approx {\dot{\gamma }}_{\text{thick}}\sim v^{\alpha }$ at low v. The exponent α decreases with increasing $p_{0}$, with α≈2.0 at $p_{0}=4.0$. In the fluid phase, $p_{0}>p_{0}^{*}$; therefore, *any* level of activity v, however small, is sufficient to restore Newtonian response $\sigma =\eta \dot{\gamma }$ in quasistatic shear $\dot{\gamma }\to 0$, as $\dot{\gamma }<{\dot{\gamma }}_{\text{thin}}\approx {\dot{\gamma }}_{\text{thick}}\sim v^{\alpha }$. This finding notably contrasts with the (zero-activity, zero-shear) solid phase, $p_{0}<p_{0}^{*}$, where a finite level of activity $v=v_{c}\left(p_{0}\right)$ is needed to give Newtonian behavior in slow shear, $\dot{\gamma }\to 0$.

**FIG. 6.**
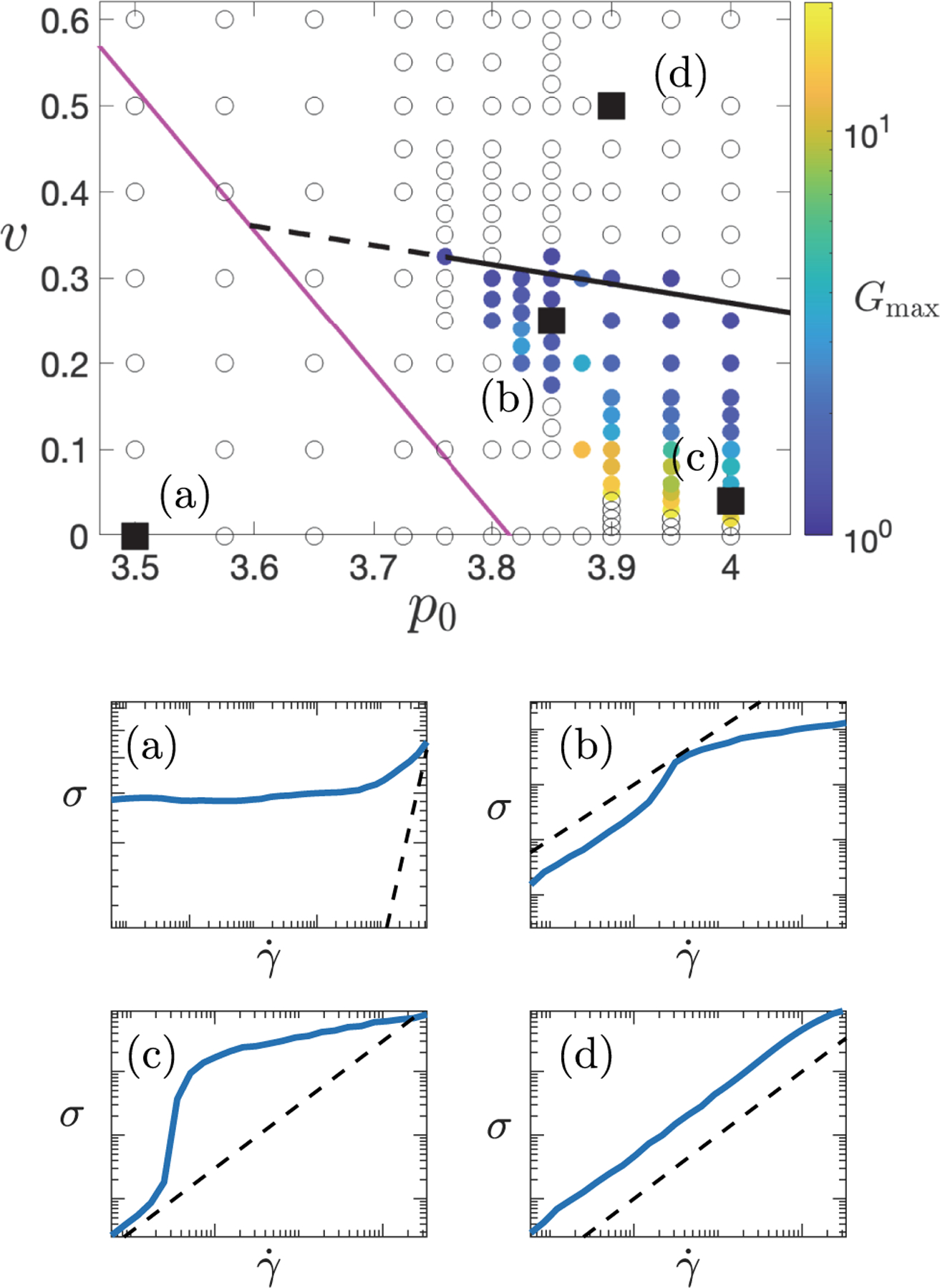
Phase diagram showing different regimes of flow behavior for different values of $v,p_{0}$, with a representative flow curve in each regime. In the top panel, colored symbols indicate maximum logarithmic slope $G_{max}$ of the flow curve at any $v,p_{0}$, provided $G_{max}>1+\epsilon$ with ϵ=0.2, designated as the criterion for shear thickening. Open circles have $G_{max}<1+\epsilon$ and no thickening. Black solid line: linear fit to $v=v\left(p_{0}\right)$ at which thickening is lost. Dashed line: extrapolation of black solid line left to meet magenta line. The magenta line is the same as in Fig. 3. Panels (a)–(d) show representative flow curves at the $v,p_{0}$ values indicated: (a) yield stress, (b) CST, (c) DST, and (d) Newtonian.

Having examined the shear rate at which shear thickening (if present) arises in the flow curve for any pairing of $p_{0},v$ values, we finally consider which flow curves indeed show shear thickening. To do so, we define $G_{max}$ to be the maximum of the logarithmic gradient $G=dlog\sigma /dlog\dot{\gamma }$ across each flow curve and plot in Fig. 6 a color map of values of $G_{max}$ that exceed 1+ϵ with ϵ=0.2, taking this as the minimal threshold for shear thickening. Values of $p_{0},v$ for which the flow curve does not meet this threshold are shown as white open symbols. As can be seen, very strong shear thickening (large $G_{max}$) arises at high $p_{0}$ and low v: This is the regime of DST, where the value of $G_{max}$ is limited only by the resolution of $\dot{\gamma }$ values simulated. As v increases at fixed $p_{0}$, we see a crossover to more moderate CST before thickening is lost at high v. The black solid line shows a linear fit to the threshold at which thickening is lost.

The apparent loss of shear thickening at fixed v with decreasing $p_{0}$ in Fig. 6 is worthy of comment. Towards the left-hand edge of the regime of colored symbols, the shear rate ${\dot{\gamma }}_{\text{thick}}$ that marks the onset of thickening decreases, approaching the minimum shear rate that we can feasibly simulate. Were we able to simulate arbitrarily low-shear rates, we speculate that the observed regime of thickening would in fact extend leftwards, with ${\dot{\gamma }}_{\text{thick}}\to 0$ only at the magenta line, consistent with the zero-shear viscosity η being infinite to the left of that line (Fig. 3). We have therefore continued the black solid line leftwards as a dashed line and suggest that the black and magenta lines together delineate the key rheological regimes observed in this work. Representative flow curves for each regime are shown beneath the color map in Fig. 6.

At higher shear rates, for all $p_{0},v$, we observe shear thinning arising from T1 cell rearrangement events. This finding has been seen previously in a vertex model, and it derives from an interplay of active fluctuations in vertex length with T1 transitions induced by shear [40].

## IV. DISCUSSIONS AND CONCLUSIONS

Our work points towards a framework for understanding the emergent nonlinear mechanics of biological tissue. In particular, we have shown the nonlinear shear rheology of the vertex model to be determined by an intricate interplay between the intrinsic solid-liquid transition that arises at a target cell shape $p_{0}=p_{0}^{*}\approx 3.81$ in the absence of shear or activity [42,70], with the mutually competing effects of a global external shear and local internal cell motility.

Indeed, in slow shear, $\dot{\gamma }\to 0$, a sufficiently high level of activity always ensures a liquidlike Newtonian response. The path to this liquified state as a function of increasing activity is however markedly different for values of the target cell shape $p_{0}$ in the (zero-shear, zero-activity) solid and liquid phases. In the former, a critical threshold activity level $v_{c}\left(p_{0}\right)$ is needed to induce liquefaction. In the latter, any level of activity, however small, ensures Newtonian response in quasistatic shear $\dot{\gamma }\to 0$. As the shear rate increases, however, the globally coherent effect of shear exceeds that of locally incoherent activity, inducing a resolidification transition via DST. The shear thickening behavior thus arises from the competition between the accumulation of shear strain due to driving and the dissipation due to cellular activity. On the one hand, the applied shear rate drives the formation of tension networks in the tissue. On the other, the cellular activity acts as a dissipative noisy process that remodels and relaxes the tension network.

We posit that this competition between externally imposed shear and internal cell motility can be characterized via a Péclet number, $Pe=\dot{\gamma }\tau_{f}$ [71], in which $\tau_{f}$ is the timescale for cell-cell rearrangements due to active motility. In the regime of high Péclet number, $Pe\gg 1,\dot{\gamma }\gg \tau_{f}^{-1}$, motility is insufficient to affect structural rearrangements caused by the imposed shear. As a result, the mechanical response of the tissue is dominated by the externally applied shear. Because this provides a global driving that acts in a coherent way across the entire tissue, it tends to deform cells away from their target shape, leading to solidlike behavior.

In contrast, in the regime of low Péclet number, $Pe\ll 1,\;\tau_{f}^{-1}\gg \dot{\gamma }$, the mechanics of the tissue is dominated by the active motility. Because it arises internally at the local level of individual cells, lacking any spatial coherence across the tissue, it provides a source of structural rearrangements that tend to counteract the solidifying effect of the globally coherent applied shear just described, resulting in a liquid-like response.

We propose that shear thickening occurs at a Péclet number of approximately 1, such that the threshold shear rate for shear thickening is proportional to the inverse of the characteristic timescale $\tau_{f}$. Deriving an expression for $\tau_{f}$ is not a simple task. However, we suggest that this timescale for structural rearrangement $\tau_{f}$ will be proportional to the tissue’s Newtonian viscosity η, defined as the ratio of stress to strain rate in the zero-shear rate limit of the flow curve, i.e., at low Péclet number Pe≪1.

**FIG. 7.**
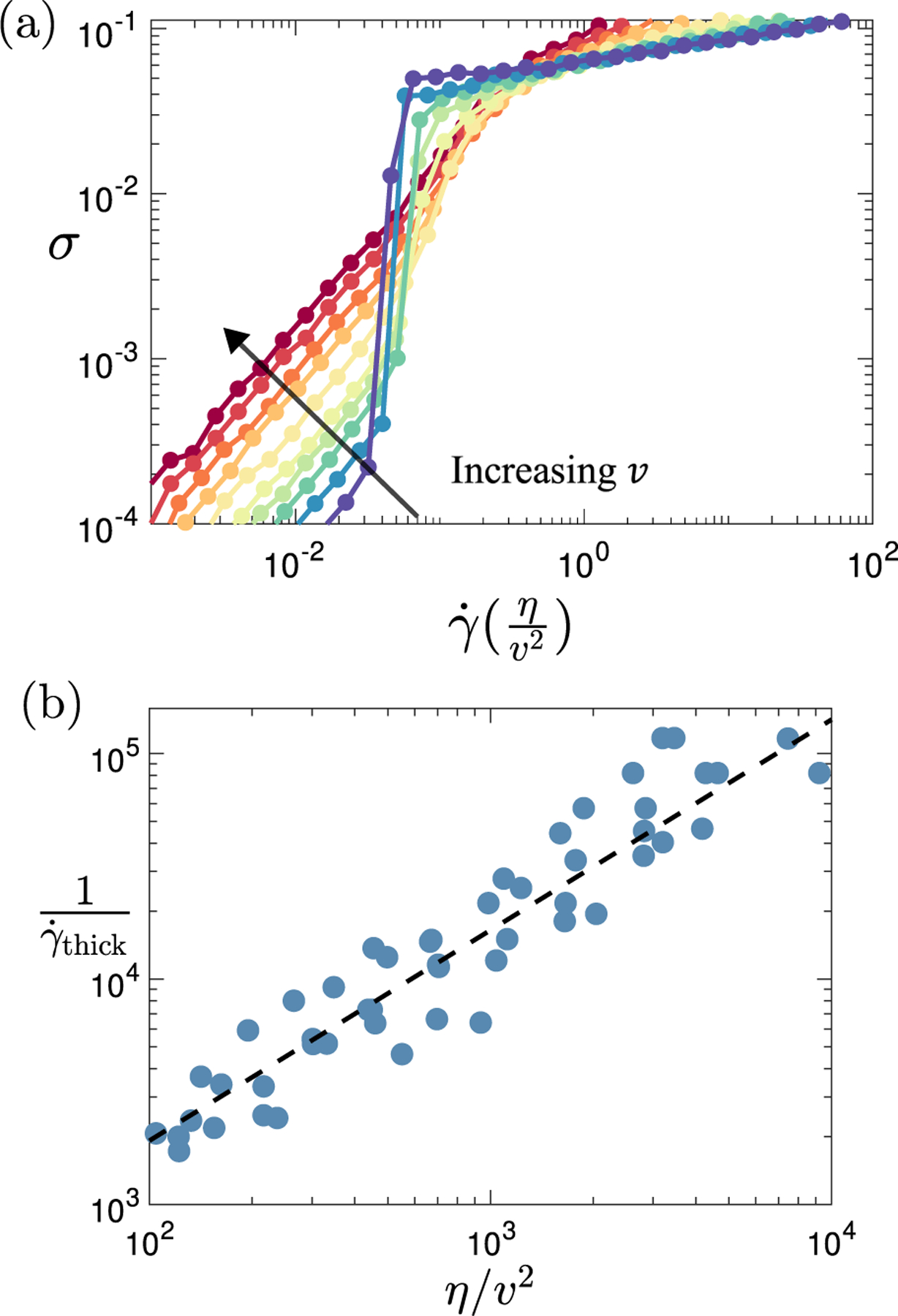
Data collapse with Péclet number. (a) Flow curves for target cell shape $p_{0}=3.90$ and activity v=0.08,0.10,0.12,0.14,0.16,0.20,0.25,0.30,0.35,0.40 (from bottom to top). Compared with flow curves shown in raw form, as, for example, in Fig. 2, the shear rate on the horizontal axis has now been rescaled, $\dot{\gamma }\to \dot{\gamma }\left(\eta /v^{2}\right)$, to demonstrate scaling collapse with respect to the location of the shear thickening transition. (b) Inverse shear rate at the shear thickening transition $1/{\dot{\gamma }}_{\text{thick}}$ plotted as a function of the scaled viscosity $\eta /v^{2}$ across the full range of values of cell shape $p_{0}$ and activity v for which a Newtonian regime and a shear thickening transition are observed in the numerically accessible flow curve. The black dashed line shows a linear scaling $1/{\dot{\gamma }}_{\text{thick}}\propto \eta /v^{2}$ as a guide to the eye.

To explore this idea, we show in Fig. 7(a) a set of flow curves for a fixed target cell shape $p_{0}=3.90$, for a range of values of the activity parameter v, now with the shear rate on the horizontal axis rescaled according to $\dot{\gamma }\to \dot{\gamma }\eta /v^{2}$. As can be seen, the location ${\dot{\gamma }}_{\text{thick}}$ of the shear thickening transition, which is different for different activity values in raw flow curves such as those shown in Fig. 2, now collapses to a single scaled shear rate, $1/{\dot{\gamma }}_{\text{thick}}\propto \eta /v^{2}$. This finding confirms that the inverse shear rate at which thickening occurs in nonlinear rheology, and thus our timescale $\tau_{f}$, is indeed proportional to the tissue’s zero-shear viscosity η. This scaling is further investigated in Fig. 7(b), which shows that the relationship $1/{\dot{\gamma }}_{\text{thick}}\propto \eta /v^{2}$ is approximately obeyed over the full range of values of v and target cell shape $p_{0}$ for which both a shear thickening transition and a Newtonian viscosity are indeed seen in the flow curve.

As just described, this rearrangement timescale $\tau_{f}$ is important in tissue mechanics because it captures the timescale for structural and stress relaxation driven by internal activity. Via this scaling argument, we have demonstrated the quantity $\tau_{f}$ to be closely related to the tissue viscosity in the limit of zero-shear rate. Importantly, this finding suggests a possible route to accessing the value of $\tau_{f}$ experimentally in tissue systems, for example, by using magnetically responsive ferrofluid microdroplets to perform quantitative spatiotemporal measurements of mechanical properties *in vivo* [72,73].

In our current model, the focus has been on the mechanical properties of individual cells and their intercellular interactions, without considering any mechanical role of the cell nucleus. Recent studies have elevated the importance of nuclear compressibility and size as factors that not only govern cell migration and rearrangement but also actively regulate cellular force generation [8,74]. In light of these findings, future research should incorporate mechanics of the cell nucleus into our existing model, which has already been shown to undergo a density-driven jamming transition [50,75]. We anticipate that the model’s rheological properties will become increasingly sensitive to nuclear packing density when the size of the nucleus is substantial relative to that of the cell. This could introduce an additional dimension of shear-thickening behavior, similar to the phenomena observed in densely packed particulate systems [63–65].

In this work, we have considered a bidisperse distribution of cell sizes. Looking ahead, it would be valuable to further include phenotypic heterogeneity by incorporating distributions of $v,p_{0},\kappa_{A}$, and $\kappa_{P}$ values, grounded in empirical measurements of single-cell properties. Previous research has demonstrated that such heterogeneity can significantly influence tissue rigidity and fluidity [68,76]. Consequently, we anticipate that the introduction of mechanical heterogeneity will give rise to intriguing and complex rheological behaviors.

As noted above, DST has been widely observed in dense granular systems with a phenomenology strikingly similar to that reported here for tissues. In each case, a sudden increase in viscosity occurs with increasing shear rate, associated with an intermittent bimodal switching of stress between low- and high-shear branches for imposed shear rates in the vicinity of the transition. However, key differences are also notable. In granular systems, DST arises via the development of frictional contacts between particles, leading to the formation of compressive force chains that percolate and bear loads across the sample [33,63]. In contrast, in tissues, we predict DST to arise when the globally coherent effects of an applied shear dominate over the local, spatially incoherent effects of cell motility, leading to the formation of tensile force chains that percolate and bear load. The possibility of vorticity banding associated with DST in tissues should be investigated in future 3D simulations that allow spatial variations in the vorticity direction, to explore the analogy with vorticity banding associated with DST in granular systems [39].

In living tissues, our model predictions can be immediately tested in the convergent extension of the Drosophila germband epithelium [77–79]. This epithelium experiences elongation along the anterior-posterior axis. During this elongation, the germband tissue is subjected to external shearing forces from neighboring structures, such as the ventral furrow, while simultaneously experiencing internal forces due to planar-polarized contractions driven by myosin II motor activity. This scenario presents a prime example of the interplay between local active forces and global deformations that is central to our theoretical framework. Analyzing the rheological response throughout this process could provide significant insights. With recent technological advances in imaging [80] and force-measurement techniques [81], such analyses are becoming increasingly attainable.

In summary, our study provides a robust framework for understanding the rheological behavior of biological tissues. Considering that nearly all living tissues are subject to a dynamic interplay between local active forces and global deformations, one of the model’s most straightforward yet far-reaching predictions is that this interplay can lead to a competition between the timescales of structural relaxation and of external driving forces. This in turn gives rise to diverse rheological responses, including discontinuous shear thickening. Consequently, we argue that these predictions are broadly applicable to a wide array of biological systems. Furthermore, as mounting evidence increasingly suggests that dense tissues operate near a jamming-unjamming transition, our theoretical contributions offer valuable insights into how tissue mechanics is modulated in proximity to these critical states.

In future work, it would be interesting to extend the concepts explored here to understand whether strongly nonlinear mechanical phenomena such as tissue fracture [23,25] and a ductile-to-brittle transition [27] are related to the tissue’s ability to shear-thicken.

The code used for this paper is available from the author upon request.

## Figures and Tables

**FIG. 8.**
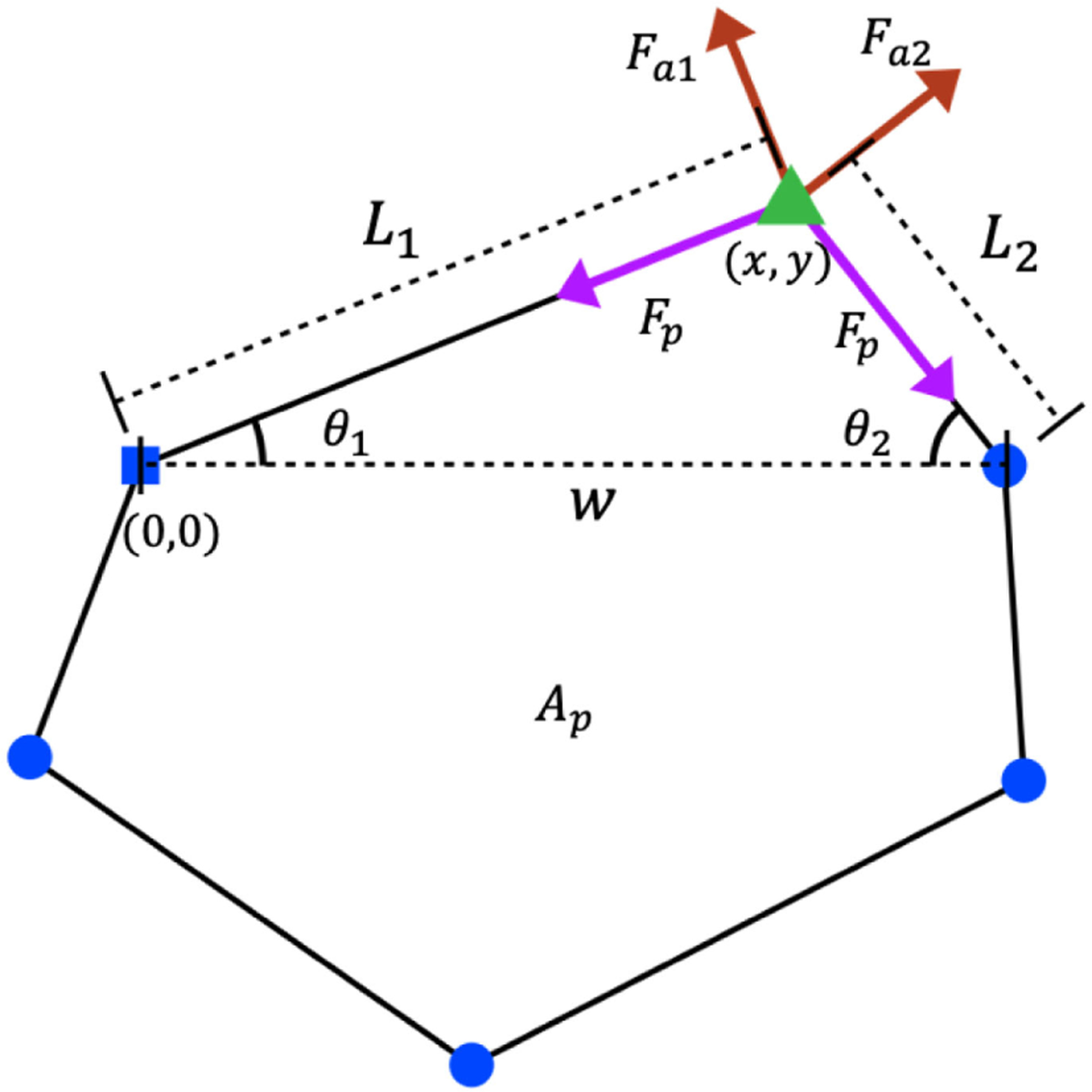
Diagram of forces acting on a single vertex from the two edges of one of the cells that meets that vertex.

**TABLE I.** Parameters of the model. Dimensions are expressed in terms of modulus (G), time (T), and length (L).

Quantity	Symbol	Dimensions	Value
Number of cells	N	1	100
Drag coefficient	ζ	GT	1 (time unit)
Edge length at initialization	L	L	1 (length unit)
Perimeter modulus	$\kappa_{\text{P}}$	G	1 (stress unit)
Area modulus	$\kappa_{\text{A}}$	GL^2^	1/2
Bidispersity ratio of the target cell area	⋯	1	1:1.4
Polarization angle diffusion	$D_{r}$	T^−1^	0.5
T1 threshold	$l_{T1}$	L	0.07
Shape factor	$p_{0}$	1	Varied
Activity	v	GL	Varied
Shear rate	$\dot{\gamma }$	T^−1^	Varied
Time step	dt	T	0.01

## APPENDIX: DERIVING VERTEX MODEL FORCES

We recall from Eq. (1) in the main text that the elastic energy of a single cell in the vertex model comprises two contributions. The first stems from the deviation of the cell’s actual area A from its target value $A_{0}$. The second stems from the deviation of the cell’s actual perimeter P from its target value $P_{0}$. The resultant force on any given vertex associated with each edge that meets that vertex then likewise comprises separate energy and perimeter contributions, as shown in Fig. 8. (Additional area and perimeter forces also arise associated with the third edge that meets the vertex from neighboring cells that are not shown.) The area force is resolved into components perpendicular to each cell edge. The perimeter force is resolved into components parallel to each cell edge. We now derive the magnitude of each of these components.

### 1. Area forces

The cell sketched in Fig. 8 can be separated into an upper triangle and a lower pentagon by the bisecting dotted line shown. With the coordinates of the vertex of interest at (x,y) and the origin (0, 0) defined to coincide with the vertex to its left, the cell area can be represented as the sum of the triangle’s area and the pentagon’s area:

(A1)
$$A=\frac{1}{2}wy+A_{p}.$$

In this equation,

(A2)
$$w=L_{1}\text{cos}\left(\theta_{1}\right)+L_{2}\text{cos}\left(\theta_{2}\right),$$

and $A_{p}$ is the area of the pentagon. The contribution of this cell to the energy in Eq. (1) can then be written

(A3)
$$E_{a}=\frac{1}{2}\kappa_{A}{\left(\frac{1}{2}wy+A_{p}-A_{0}\right)}^{2}.$$

The x and y components of the area forces can then be calculated by taking the gradient of $E_{a}$,

(A4)
$$\nabla E_{a}\;=\left[\begin{array}{c} 0\\ \frac{1}{2}\kappa_{A}w\left(A_{0}-A\right) \end{array}\right],\;=\left[\begin{array}{c} -F_{a1}\text{sin}\left(\theta_{1}\right)+F_{a2}\text{sin}\left(\theta_{2}\right)\\ F_{a1}\text{cos}\left(\theta_{1}\right)+F_{a2}\text{cos}\left(\theta_{2}\right) \end{array}\right].$$

On the second line, the x and y component forces are expressed in terms of the components $F_{a1}$ and $F_{a2}$ perpendicular to the cell edges. The x component then yields the equation

(A5)
$$0=-F_{a1}\text{sin}\left(\theta_{1}\right)+F_{a2}\text{sin}\left(\theta_{2}\right),$$

and the y component yields the equation

(A6)
$$\frac{1}{2}\kappa_{A}w\left(A_{0}-A\right)=F_{a1}\text{cos}\left(\theta_{1}\right)+F_{a2}\text{cos}\left(\theta_{2}\right).$$

Recognizing that

(A7)
$$L_{1}\text{sin}\left(\theta_{1}\right)=L_{2}\text{sin}\left(\theta_{2}\right),$$

we obtain, together with Eq. (A5),

(A8)
$$F_{a2}=F_{a1}\frac{L_{2}}{L_{1}}.$$

Substituting this into Eq. (A6) gives

(A9)
$$\frac{1}{2}\kappa_{A}w\left(A_{0}-A\right)=F_{a1}\text{cos}\left(\theta_{1}\right)+F_{a1}\frac{L_{2}}{L_{1}}\text{cos}\left(\theta_{2}\right).$$

Rearranging this equation gives

(A10)
$$F_{a1}=\frac{\frac{1}{2}\kappa_{A}wL_{1}\left(A_{0}-A\right)}{L_{1}\text{cos}\left(\theta_{1}\right)+L_{2}\text{cos}\left(\theta_{2}\right)}.$$

Substituting the expression for w from Eq. (A2) then gives

(A11)
$$F_{a1}=\frac{1}{2}\kappa_{A}L_{1}\left(A_{0}-A\right).$$

Substituting this into Eq. (A8) likewise gives

(A12)
$$F_{a2}=\frac{1}{2}\kappa_{A}L_{2}\left(A_{0}-A\right).$$

### 2. Perimeter forces

The perimeter forces can be derived using a similar method. We start by separating the cell perimeter into the contribution from the triangle, $L_{1}+L_{2}$, and the contribution from the pentagon, $P_{p}$ (this being the pentagon’s perimeter minus w):

(A13)
$$P=L_{1}+L_{2}+P_{p}.$$

In this equation,

(A14)
$$L_{1}=\sqrt{x^{2}+y^{2}}$$

and

(A15)
$$L_{2}=\sqrt{(w-x{)}^{2}+y^{2}.}$$

The contribution of this cell to the energy in Eq. (1) can then be written

(A16)
$$E_{p}=\frac{1}{2}\kappa_{p}{\left(\sqrt{x^{2}+y^{2}}+\sqrt{(w-x{)}^{2}+y^{2}}+P_{p}-P_{0}\right)}^{2}.$$

The x and y components of the perimeter forces can then be calculated by taking the gradient of $E_{p}$:

(A17)
$$\nabla E_{p}\;=\left[\begin{array}{c} \kappa_{p}\left(\frac{x}{L_{1}}-\frac{w-x}{L_{2}}\right)\left(P-P_{0}\right)\\ \kappa_{p}\left(\frac{y}{L_{1}}+\frac{y}{L_{2}}\right)\left(P-P_{0}\right) \end{array}\right],\;=\left[\begin{array}{c} F_{P}\left(\text{cos}\left(\theta_{1}\right)-\text{cos}\left(\theta_{2}\right)\right)\\ F_{P}\left(\text{sin}\left(\theta_{1}\right)+\text{sin}\left(\theta_{2}\right)\right) \end{array}\right].$$

On the first line of this equation, we have substituted the expressions for $P,L_{1}$, and $L_{2}$ from Eqs. (A13)–(A15). Further recognizing that

(A18)
$$\frac{x}{L_{1}}=\text{cos}\left(\theta_{1}\right),\;\frac{w-x}{L_{2}}=\text{cos}\left(\theta_{2}\right),$$

together with

(A19)
$$\frac{y}{L_{1}}=\text{sin}\left(\theta_{1}\right),\;\frac{y}{L_{2}}=\text{sin}\left(\theta_{2}\right),$$

we finally obtain

(A20)
$$F_{P}=\kappa_{p}\left(P-P_{0}\right).$$
